# Identification of Key miRNAs in the Treatment of Dabrafenib-Resistant Melanoma

**DOI:** 10.1155/2021/5524486

**Published:** 2021-04-05

**Authors:** Guangyu Gao, Zhen Yao, Jiaofeng Shen, Yulong Liu

**Affiliations:** ^1^Department of Nuclear Accident Medical Emergency, The Second Affiliated Hospital of Soochow University, Suzhou 215004, China; ^2^Department of Oncology, The Second Affiliated Hospital of Soochow University, Suzhou 215004, China; ^3^State Key Laboratory of Radiation Medicine and Protection, School of Radiation Medicine and Protection, Soochow University, Suzhou 215123, China; ^4^Collaborative Innovation Center of Radiological Medicine of Jiangsu Higher Education Institutions, Suzhou 215123, China

## Abstract

Dabrafenib resistance is a significant problem in melanoma, and its underlying molecular mechanism is still unclear. The purpose of this study is to research the molecular mechanism of drug resistance of dabrafenib and to explore the key genes and pathways that mediate drug resistance in melanoma. GSE117666 was downloaded from the Gene Expression Omnibus (GEO) database and 492 melanoma statistics were also downloaded from The Cancer Genome Atlas (TCGA) database. Besides, differentially expressed miRNAs (DEMs) were identified by taking advantage of the R software and GEO2R. The Database for Annotation, Visualization, and Integrated Discovery (DAVID) and FunRich was used to perform Gene Ontology (GO) and Kyoto Encyclopedia of Genes and Genomes (KEGG) pathway enrichment analysis to identify potential pathways and functional annotations linked with melanoma chemoresistance. 9 DEMs and 872 mRNAs were selected after filtering. Then, target genes were uploaded to Metascape to construct protein-protein interaction (PPI) network. Also, 6 hub mRNAs were screened after performing the PPI network. Furthermore, a total of 4 out of 9 miRNAs had an obvious association with the survival rate (*P* < 0.05) and showed a good power of risk prediction model of over survival. The present research may provide a deeper understanding of regulatory genes of dabrafenib resistance in melanoma.

## 1. Introduction

By 2018, 1,762,450 new tumor patients are been made definite diagnosed and 606,880 among them died in the United States [1]. Of them, melanoma is the most aggressive type of skin tumor, takes up 10% of all skin cancers, but causes more than 80% of skin carcinoma-related deaths [2]. In 2019, approximately 96,480 individuals may be diagnosed with melanoma, and 7230 may have died of the disease. Although the overall incidence of cancer has declined in men and remained stable in women, the incidence of cutaneous melanoma in the United States has continued to rise over the past decade [1]. Based on conventional chemotherapy, melanoma does not respond well to treatment, leading to a 5-year survival rate of only 15% [3]. Molecular changes associated with sun exposure [4] or DNA methylation [5] are thought to be related to the development of melanoma. Besides, the mitogen-activated protein kinase (MAPK) pathway and gene mutations in the MEK/RAS/RAF/ERK have been found [6] and have offered new drug treatment targets. Among the second generation of selective BRAFV600E inhibitors, dabrafenib is the first drug authorized for the targeted treatment of unresectable melanoma [7]. Although the efficacy and tumor control effect of BRAF inhibitors are significant, the persistence of the efficacy is limited due to drug resistance, and signs of disease progression can be seen within 6-8 months after the start of treatment [8, 9].

microRNA (miRNA) is a highly conserved and short noncoding RNA molecule that occurs naturally in plant and animal genomes. They usually bind to the untranslated 17′-mRNA by regulating the length of the utr-3 region. Studies have shown that thousands of human protein-coding genes are regulated by miRNAs, indicating that miRNAs are the “main regulators” of many important biological processes [10]. Although miRNA has only 20 nucleotides, it plays an important role in gene expression by regulating a large number of target genes [11]. For example, Zhang et al. found that miR-129-5p can suppress lung cancer cell viability and invasion, which may occur via the modulating of MCRS1, E-cadherin, and vimentin expression [12]. Zhang et al. found that low miR-133 expression was a common event and correlated with worse clinical outcomes in acute myeloid leukemia, suggesting that serum miR-133 might serve as a promising indicator for the early detection and prognosis evaluation of AML [13]. Xu et al. also reported that miRNA-100 inhibits human bladder urothelial carcinogenesis by directly targeting mTOR [14]. Besides, different mechanisms of BRAF inhibitor resistance in melanoma have been described: epigenetic [15], genomic [16], and phenotypic [17] transformation produces many changes leading to acquired, internal, or adaptive resistance. However, it still needs to be further explored.

In the current study, microarray data for GSE117666 and melanoma sample data in the TCGA database facilitated the investigation of differently expressed miRNAs in dabrafenib-sensitive and dabrafenib-resistant melanoma. The functions of the target mRNAs were assessed by using GO annotation, KEGG, PPI network, and the relationship between dabrafenib-resistant miRNAs and the overall survival of patients with cancer. In summary, we performed this study to find the key miRNAs and mRNAs of medicine resistance and to discover potential new tumor therapy targets to reduce dabrafenib resistance.

## 2. Materials and Methods

### 2.1. Microarray Data

**Figure 1:**
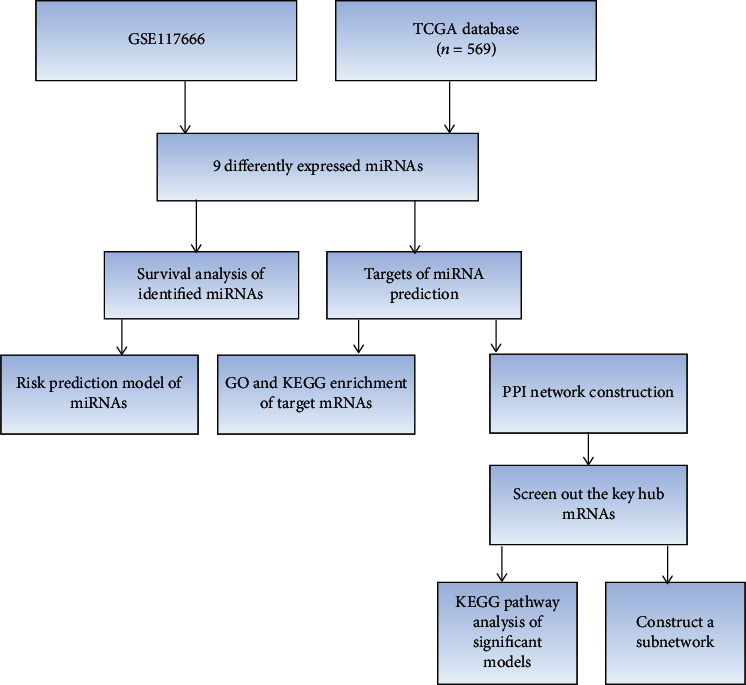
Flow chart of this study. GO: Gene Ontology; miRNA: microRNA; mRNA: messenger RNA; PPI: protein-protein interaction; KEGG: Enriched Kyoto Encyclopedia of Genes and Genomes.

The GEO (https://www.ncbi.nlm.nih.gov/gds) database is a gene expression database created and maintained by NCBI. It was founded in 2000 and contains high-throughput gene expression data submitted by research institutions around the world. In our research, gene expression profile data (GSE117666) was obtained from GEO. Three dabrafenib-resistant melanoma cells and three dabrafenib-sensitive cell lines were included. The array data were acquired from the Affymetrix Multispecies miRNA-3 Array [GPL16384; transcript (gene) version]. Besides, the flow of this study is shown in Figure 1.

### 2.2. DEMs Analysis

We used the R software to compare the two groups of samples. Furthermore, ∣log2FC | ≥2 and *P* < 0.05 were set up as cut-off criteria, and if the statistics according to our criteria, obvious statistical differences will be considered [18].

### 2.3. Targets of miRNA Prediction

DEMs were achieved by the method mentioned above. miRWalk1.0 (http://mirwalk.umm.uni-heidelberg.de/) is a fully documented, freely available database that provides the largest set of predictive, experimentally proven miRNA target interactions in a variety of novel ways.

### 2.4. Functional and Pathway Enrichment Analysis

KEGG pathway analysis and GO functional analysis of the DEGs we identified were performed by using FunRich software. It is a stand-alone software tool used mainly for functional enrichment and interaction network analysis of genes and proteins. Besides, the results of the analysis can be depicted graphically in the form of Venn, Bar, Column, Pie, and Doughnut charts. GO analysis was divided into the cellular component (CC), biological process (BP), and molecular function (MF), and a *P* value < 0.05 was thought that there was a statistical difference. We also used ClueGO for KEGG pathway analysis. It is a Cytoscape plug-in that visualizes the nonredundant biological terms for large clusters of genes in a functionally grouped network. The identifiers can be uploaded from a text file or interactively from a network of Cytoscape.

### 2.5. Protein-Protein Interaction Network Analysis

To analyze the connection among proteins, target mRNAs were uploaded to Search Tool for the Retrieval of Interacting Genes (STRING, https://string-db.org/), a database covering 9,643,763 proteins from 2,031 organisms, and the result whose minimum interaction score was 0.4 was visualized in Cytoscape [19]. Furthermore, the Molecular Complex Detection (MCODE) was used to find obvious modules based on the constructed PPI networks with the criteria of degree cut-off = 2, node density cut-off = 0.1, and node score cut-off = 0.2, and hub genes were exported.

### 2.6. Analysis of mRNAs Expression in Melanoma

Hub gene expression in melanoma tissues and normal tissues was extracted from the human protein atlas (http://www.proteinatlas.org). The Human Protein Atlas is a Swedish-based program initiated in 2003 to map all the human proteins in cells, tissues, and organs using an integration of various omics technologies, including antibody-based imaging, mass spectrometry-based proteomics, transcriptomics, and systems biology. Gene expressions we selected were determined through analysis of TCGA databases, which are available through TCGAportal (http://www.tcgaportal.org).

### 2.7. Analysis of the miRNAs and Their Relationship with Melanoma Prognosis

The Kaplan-Meier Plotter (http://www.kmplot.com/) is capable to assess the effect of 54k genes (mRNA, miRNA, protein) on survival in 21 cancer types including breast (*n* = 6,234), ovarian (*n* = 2,190), lung (*n* = 3,452), and gastric (*n* = 1,440) cancer. Sources for the databases include GEO, EGA, and TCGA. The primary purpose of the tool is a meta-analysis-based discovery and validation of survival biomarkers. [20]. Each miRNA that was selected would then be entered into the online tool to evaluate the survival of patients with melanoma. Meanwhile, we extracted HRs with 95% CI of every identified miRNA and draw a Forest plot using the Stata 14.0 software.

### 2.8. Independent Prognostic Ability of the miRNA Signature

To learn more about the association between identified miRNAs and the prognosis of melanoma patients, we performed a risk prediction model. 492 melanoma statistics were also downloaded from the TCGA database. The 3-year and 5-year AUC dependent on the ROC curve was calculated using the “survivalROC” software package to evaluate the predictive ability of the identified miRNAs. A block diagram was drawn to show the risk score of the model.

## 3. Results

### 3.1. Identification of the miRNAs between Dabrafenib Sensitive and Dabrafenib Resistant Melanoma Cells

The R software was used to research the gene expression profiles from the GSE117666. It highlighted the DEGs between GSM3305847, GSM3305851, GSM3305852 (dabrafenib-sensitive), GSM33305848, GSM3305849, and GSM3305850 (acquired dabrafenib-resistant) melanoma cells. According to the cut-off criteria (*P* < 0.05 and ∣log2FC | ≥2), 30 DEMs were selected. After researching 492 samples of the TCGA database, 72 DEMs were identified and 9 of them exist in both filter results which were consisted of 6 downregulated and 3 upregulated miRNAs. The result is shown in Table 1.

### 3.2. Target Prediction and GO Analysis

**Figure 2:**
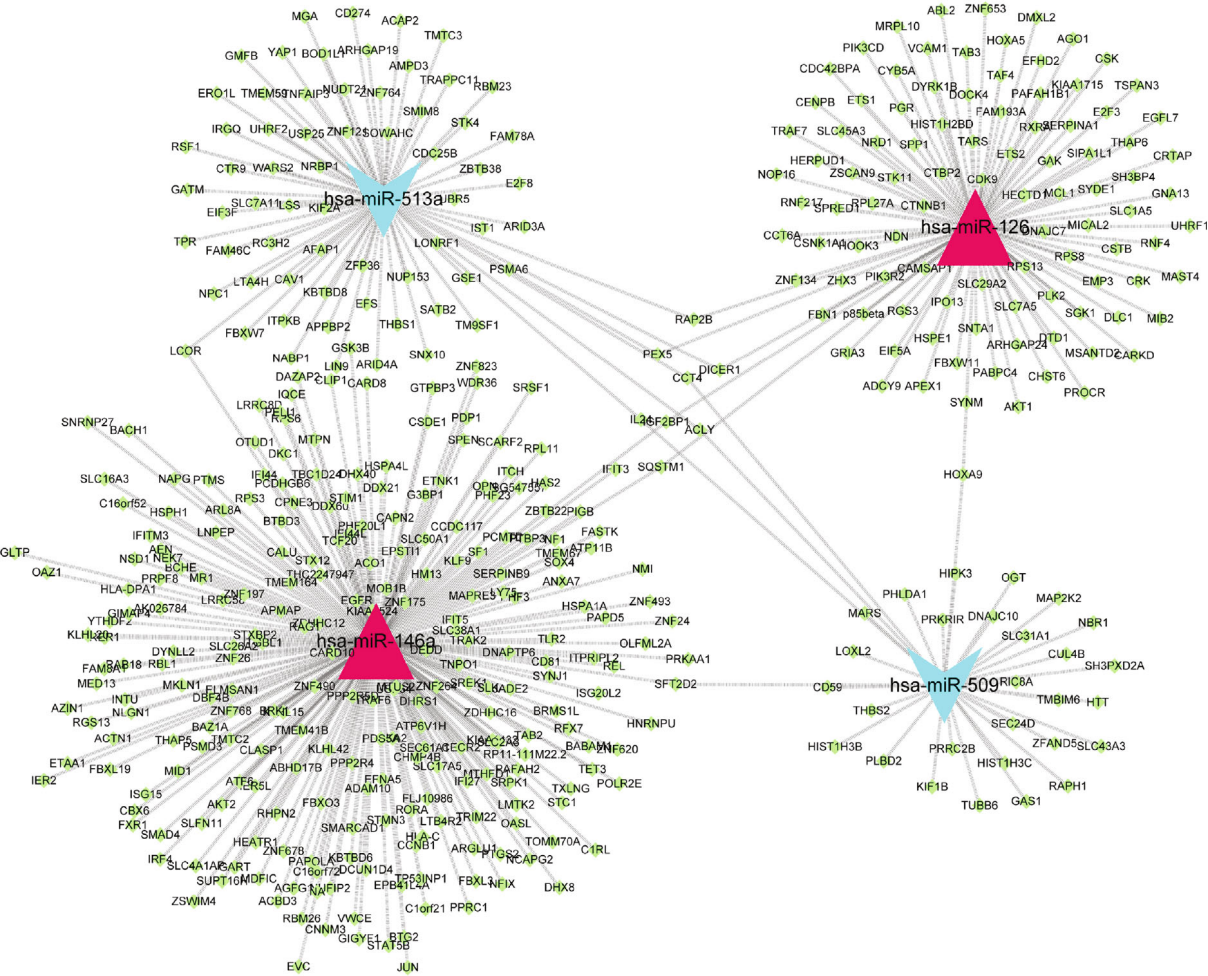
The interactions between differentially expressed miRNAs and target mRNAs.

**Figure 3:**
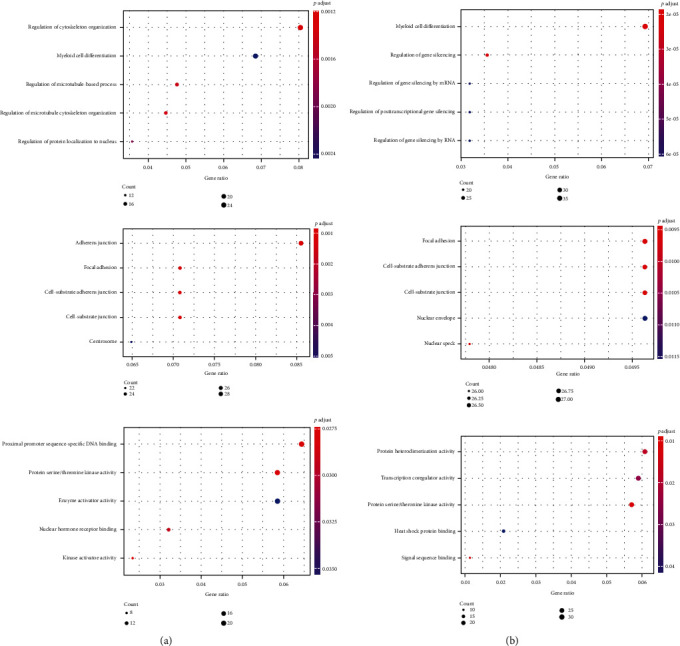
Differentially expressed mRNAs analyzed by GO enrichment. CC: cellular component; MF: molecular function; BP: biological process.

The target mRNAs of those 9 DEMs were downloaded from two miRNA target prediction websites (targetscan and miRDB). 872 mRNAs were identified after filtering. The network between miRNAs (2 upregulated miRNAs and 2 downregulated miRNAs) and target mRNAs was shown in Figure 2. To learn more about the function of these mRNAs, these mRNAs were uploaded into FunRich to perform GO and KEGG analysis. In the CC ontology, DEGs were enriched in “Cytoplasm”, “Plasma membrane”, and “Nucleus”. In the BP ontology, DEGs were enriched in “Transport”, “Cell growth and/or maintenance”, and “Regulation of nucleobase, nucleoside, nucleotide, and nucleic acid metabolism”. In the MF ontology, DEGs were enriched in “Protein serine/threonine kinase activity”, “Cell adhesion molecule activity”, and “Guanyl-nucleotide exchange factor activity” (Figure 3).

### 3.3. KEGG Pathways of DEMs

**Figure 4:**
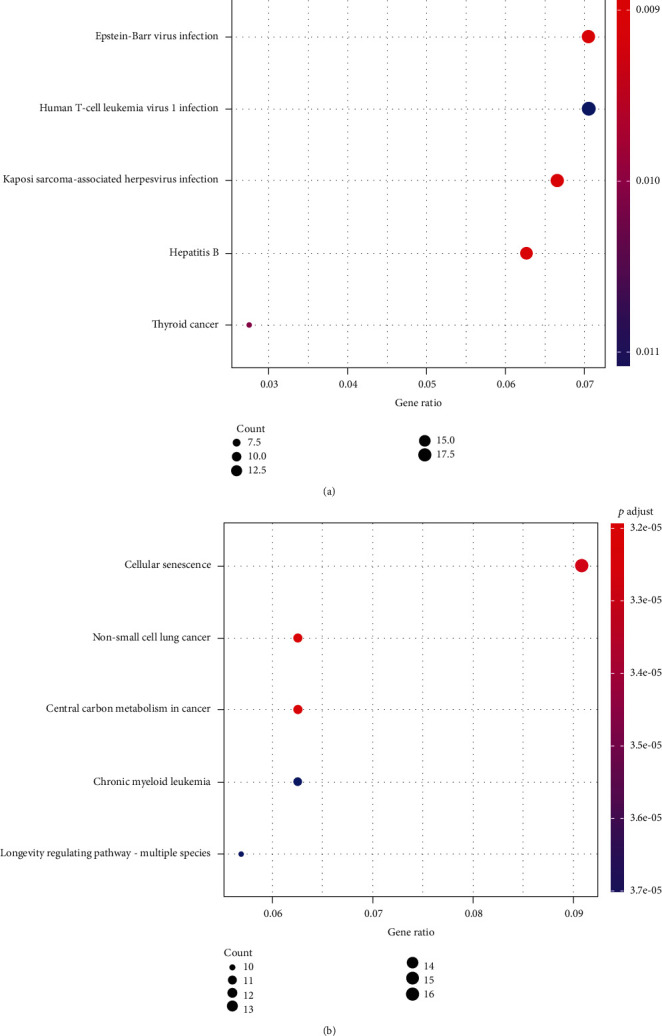
KEGG pathway enriched by up- and downregulated mRNAs.

By using ClueGO, we identified several KEGG significant enriched pathways. DEMs were enriched in “Pathways in cancer”, “Proteoglycans in cancer”, “microRNAs in cancer”, “Wnt signaling pathway”, “Central carbon metabolism in cancer, and “Cytokine-cytokine receptor interaction” (Figure 4).

### 3.4. Construction of a Protein-Protein Interaction (PPI) Network

**Figure 5:**
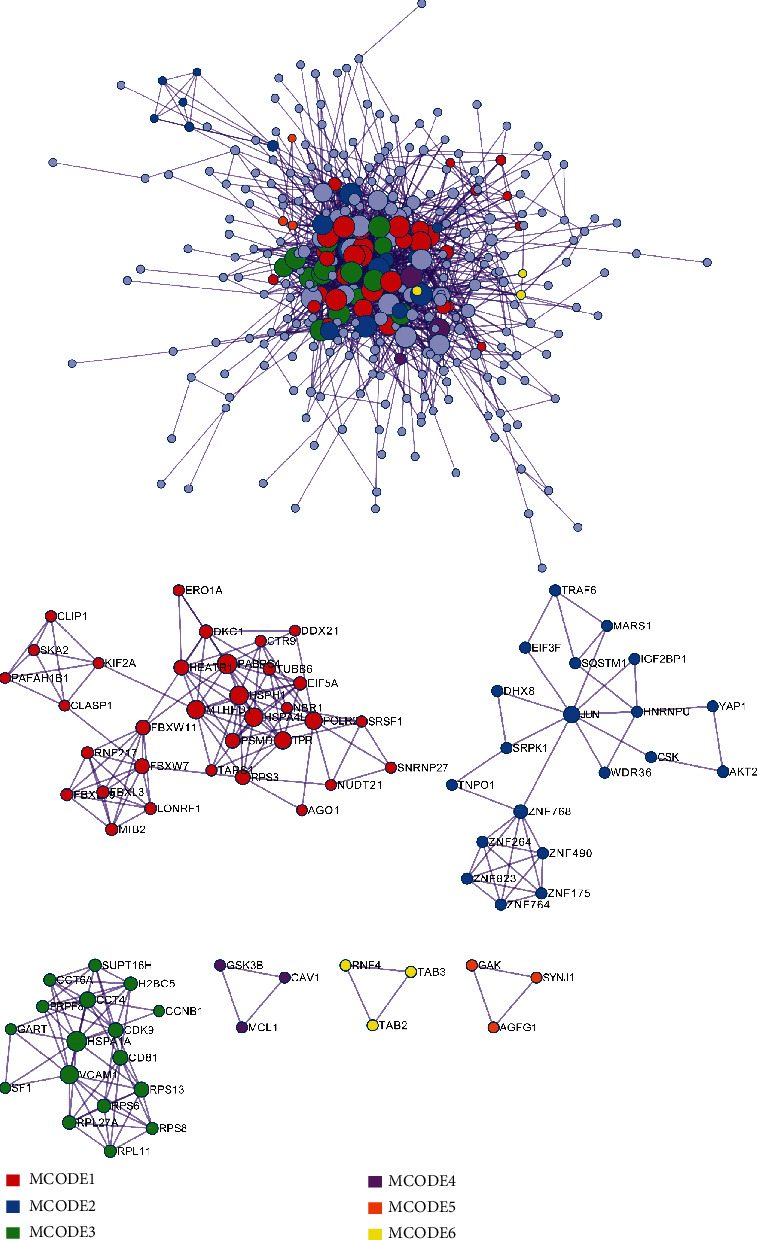
PPI network and identification of MCODEs.

**Figure 6:**
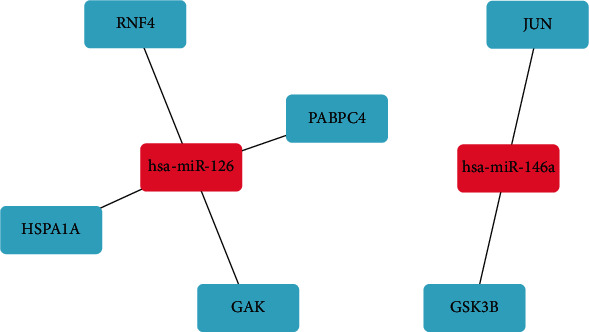
miRNA mRNA regulatory pairs from the 6 hub miRNA targets and their regulated miRNAs.

872 genes were inputted into the Metascape website to get interactive data. Then, if the combined score was ≥0.7, we would choose identified mRNAs to build a PPI network (Figure 5). In the PPI network, 6 modules, including PABPC4, JUN, HSPA1A, GSK3B, RNF4, and GAK were identified. The outcomes of the KEGG pathway between modules were related to “Separation of Sister Chromatids”, “Osteoclast differentiation”, “Infectious disease”, “Positive regulation of apoptotic signaling pathway”, “Clathrin-mediated endocytosis”, and “Intracellular receptor signaling pathway” (Table 2). Based on the 6 key mRNAs and target miRNAs, we performed a miRNA-mRNA network (Figure 6), and it may provide a series of promising treatment targets and enlightened us on the further investigations of the molecular mechanisms.

### 3.5. Analysis of the Expression of the Key Genes in Normal Tissues and Melanoma Tissues

**Figure 7:**
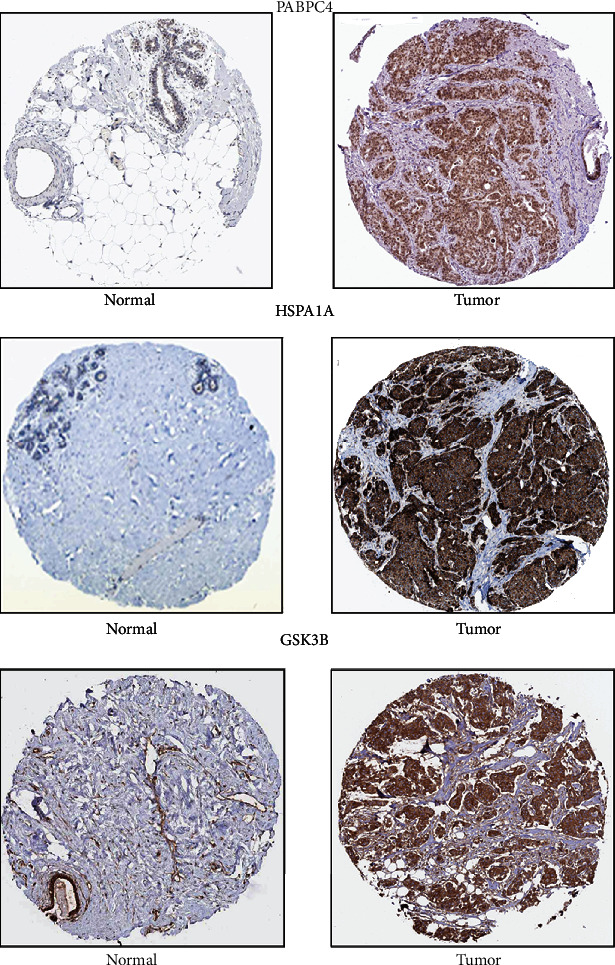
Genes expression of in human melanoma specimens. PABPC4, HSPA1A, and GSK3B expression in normal tissue and melanoma specimens.

Based on the human protein atlas, the expression of target genes was researched. PABPC4, HSPA1A, and GSK3B were identified from 6 key mRNAs. After entering them into the database, we found that three mRNAs have a positive strong expression in melanoma samples and a negative weak expression in normal samples (Figure 7).

### 3.6. Analysis of the miRNAs and Their Relationship with Melanoma Prognosis

**Figure 8:**
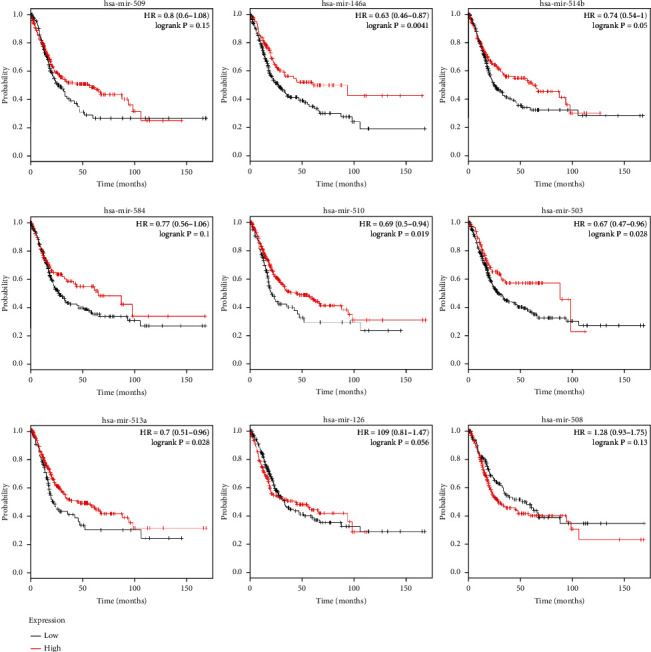
The association between miRNAs and melanoma prognosis.

**Figure 9:**
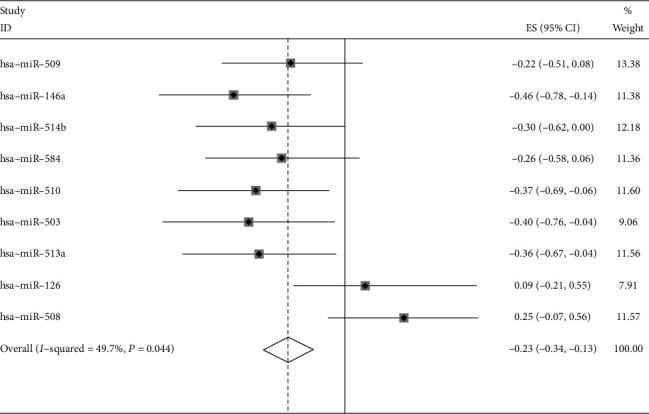
Forest plot demonstrating the association between identified miRNAs expression and the survival of humans with melanoma.

The Kaplan-Meier Plotter was utilized to study the prognosis of patients with melanoma-related to miRNAs we selected. After uploaded 9 miRNAs, we got 9 survival graphs. The results indicated that overexpression of hsa-miR-510, hsa-miR-503, and hsa-miR-513a (Figure 8) was related to improved overall survival in patients with melanoma. However, the expression level of hsa-miR-509, hsa-miR-146a, hsa-miR-514b, hsa-miR-584, hsa-miR-126, and hsa-miR-508 may have no obvious association with the OS. The forest plot showed in more detail the relationship between miRNAs expression and the survival and prognosis of patients (Figure 9). This suggested that the identified miRNAs may be potential targets for dabrafenib resistance in melanoma.

### 3.7. Evaluation of the 9-miRNA Signature for over Survival

**Figure 10:**
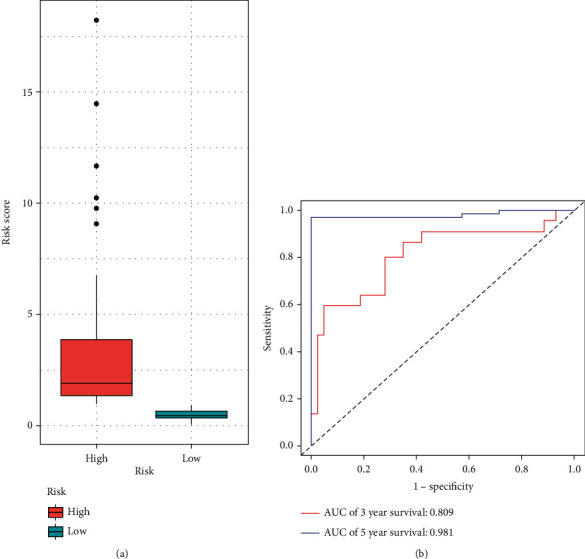
Risk prediction model of 9 identified miRNAs. (a) Box diagram of the risk score. (b) The AUC of 3 years and 5 years curve.

The AUC of 3 years survival for the 9-miRNA signature achieved 0.809 and the AUC of 5 years survival achieved 0.981, which proved that the model has good performance in predicting the survival risk of melanoma patients. Besides, the box diagram also proves our conclusion (Figure 10).

## 4. Discussion

In recent years, the incidence and mortality of malignant melanoma are increasing year by year. Compared with other solid tumors, the age of death is lower. In addition to early surgical resection, malignant melanoma lacks specific treatment and has a poor prognosis. Therefore, the early diagnosis and treatment of malignant melanoma are extremely important. In this article, GSE117666 and 492 melanoma statistics from the TCGA database were downloaded for further study. Melanoma statistics from the TCGA database were searched from the GEO and TCGA database. 9 DEMs (hsa-miR-510, hsa-miR-503, hsa-miR-513a, hsa-miR-509, hsa-miR-146a, hsa-miR-514b, hsa-miR-584, hsa-miR-126, and hsa-miR-508) were selected by combining two screening results. Among them, certain miRNAs have been shown to affect tumor proliferation, migration, and prognosis. For example, a previous study reported miR-510 promotes thyroid cancer cell proliferation, migration, and invasion through suppressing lncRNA SNHG15 [21]; it also serves as a prognostic biomarker, or as a potential therapeutic target, in cutaneous melanoma patients [22]. hsa-miR-146a controls Immune Response in the Melanoma Microenvironment and may provide a new therapeutic strategy to improve the current management of patients with melanoma [23]. Another study also reported hsa-miR-126 downregulation contributes to dabrafenib acquired resistance in melanoma by upregulating ADAM9 and VEGF-A [24]. Besides, it may play a tumor suppressor role by directly regulating ADAM9 and MMP7 in melanoma [25].

To understand the regulatory mechanism of the 9 miRNAs in melanoma, we chose the intersection of two screening results from MiRWalk. 872 mRNAs were identified after filtering. Function annotation indicated that these miRNAs were primarily related to “Cytoplasm”, “Plasma membrane”, “Nucleus”, “Transport”, “Cell growth and/or maintenance”, “Regulation of nucleobase, nucleoside, nucleotide and nucleic acid metabolism”, “Protein serine/threonine kinase activity”, “Cell adhesion molecule activity”, and “Guanyl-nucleotide exchange factor activity”. KEGG pathway analysis of DEGs revealed involvement in “Pathways in cancer”, “Proteoglycans in cancer”, “microRNAs in cancer”, “Wnt signaling pathway”, “Central carbon metabolism in cancer”, and “Cytokine-cytokine receptor interaction”. According to previous studies, these signaling pathways participate in a diverse array of important cellular processes, including the survival, proliferation, differentiation, and activation of different cell types [26–28].

Besides, PPI network analysis indicated that 6 hub genes including PABPC4 (Poly(A) Binding Protein Cytoplasmic 4), JUN (Jun Proto-Oncogene, AP-1 Transcription Factor Subunit), HSPA1A (Heat Shock Protein Family A (Hsp70) Member 1A), GSK3B (Glycogen Synthase Kinase 3 Beta), RNF4 (Ring Finger Protein 4), and GAK (cyclin G associated kinase) may be used as new targets in dabrafenib-resistant melanoma which had higher degrees of interaction. PABPC4, a protein kinase, may be a valuable source of biomarkers for response to docetaxel-resistance prostate cancer therapy [29]. As for HSPA1A, a kind of inducible heat shock protein promotes tumor cell growth and survival [30]. It also adjusts the transfer process, including EMT and migration, and seems to be destroyed by the Hsp70-dependent heterocomplexes of E-cadherin/catenins, which act as an anchor between neighboring cells [31]. GSK3B, glycogen synthase kinase 3, was identified as novel tivantinib targets [32]. It is also related to the regulation of melanogenesis, and drug inhibition can increase melanogenesis through Wnt/beta-catenin pathway activation [33]. As for RNF4, it is a SUMO-targeted ubiquitin ligase that stabilizes a specific group of oncoproteins. It enhances tumor protein activity and acts as a positive feedback agonist for Wnt and Notch pathways. RNF4 is also necessary for the survival of cancer cells, and its overexpression is related to the poor prognosis of some cancer patients [34]. GAK (Cyclin G Associated Kinase), a protein expressed ubiquitously in various tissues, is identified as the off-target responsible for intracellular melanin accumulation. It also represents a new possible target for the prevention and treatment of irregular pigmentation by the melanogenic biosynthetic pathway [35].

Furthermore, the relationship between the 9 miRNAs and the overall survival curves for patients with melanoma revealed that overexpression of hsa-miR-510, hsa-miR-503, and hsa-miR-513a was related to improved overall survival. However, the expression level of hsa-miR-509, hsa-miR-146a, hsa-miR-514b, hsa-miR-584, hsa-miR-126, and hsa-miR-508 may have no significant relationship with the overall survival of patients. Meanwhile, evaluation of the 9-miRNA signature for overall survival by the ROC curve displayed better predictive ability.

At present, with the development of targeted therapy, more and more attention has been paid to tumor drug resistance. Therefore, it is necessary to find new biomarkers and treatment methods for patients with drug resistance. Our findings concluded that these miRNAs may act as an important role in dabrafenib-resistant melanoma. However, all of our data are obtained from GEO and TCGA database through bioinformatics analysis, and the number of relevant samples is limited; further data analysis and clinical trials are needed to verify.

## 5. Conclusion

Our study not only explored certain mechanisms of dabrafenib-resistance in melanoma but also constructed a significant 9-miRNA risk prediction model for overall survival. Bioinformatics methods were used to select the DEMs in dabrafenib-resistant melanoma cells. These findings significantly improve the understanding of the cause and underlying molecular events in melanoma, and the candidate genes and pathways could be used as therapeutic targets.

## Figures and Tables

**Table 1:** Key differently expressed miRNAs of joint screening from GSE117666 and TCGA database.

ID	*P* value	Log FC
hsa-miR-509p	0.000011	-5.21303
hsa-miR-146a	0.000033	5.17469
hsa-miR-514b	0.000108	-3.48325
hsa-miR-584	0.000121	3.317673
hsa-miR-510	0.000138	-2.69851
hsa-miR-503	0.000901	-2.30348
hsa-miR-513a	0.001138	2.818463
hsa-miR-126	0.001620	-2.45363
hsa-miR-508	0.019126	-2.10383

**Table 2:** KEGG enrichment analysis of hub target mRNAs.

MCODE	ID	Description	Log10(*P*)
MCODE_1	hsa05165	Separation of sister chromatids	-8.2
MCODE_2	hsa04722	Osteoclast differentiation	-5.4
MCODE_3	hsa04919	Infectious disease	-10.1
MCODE_4	hsa04218	Positive regulation of apoptotic signaling pathway	-6.4
MCODE_5	hsa05163	Clathrin-mediated endocytosis	-6.7
MCODE_6	hsa05205	Intracellular receptor signaling pathway	-5.8

## Data Availability

All data included in this study are available from the corresponding author upon request.
